# Toward evidence-based communication on overweight body mass index and mortality

**DOI:** 10.1186/s12916-024-03402-2

**Published:** 2024-05-01

**Authors:** Maya B. Mathur, Vandana S. Mathur

**Affiliations:** 1 Quantitative Sciences Unit and Department of Pediatrics, Stanford University, Porter Drive, Palo Alto, CA 94304 USA; 2 MathurConsulting LLC, Woodside, CA USA

**Keywords:** Obesity, Physician–patient communication, Evidence-based medicine, Public health

## Abstract

**Background:**

Reducing overweight and obesity has been a longstanding focus of public health messaging and physician–patient interactions. Clinical guidelines by major public health organizations describe both overweight and obesity as risk factors for mortality and other health conditions. Accordingly, a majority of primary care physicians believe that overweight BMI (even without obesity) strongly increases mortality risk.

**Main points:**

The current evidence base suggests that although both obese BMI and underweight BMI are consistently associated with increased all-cause mortality, overweight BMI (without obesity) is not meaningfully associated with increased mortality. In fact, a number of studies suggest modest protective, rather than detrimental, associations of overweight BMI with all-cause mortality. Given this current evidence base, clinical guidelines and physician perceptions substantially overstate all-cause mortality risks associated with the range of BMIs classified as “overweight” but not “obese.” Discrepancies between evidence and communication regarding mortality raise the question of whether similar discrepancies exist for other health outcomes.

**Conclusions:**

Health communication that inaccurately conveys current evidence may do more harm than good; this applies to communication from health authorities to health practitioners as well as to communication from health practitioners to individual patients. We give three recommendations to better align health communication with the current evidence. First, recommendations to the public and health practitioners should distinguish overweight from obese BMI and at this time should not describe overweight BMI as a risk factor for all-cause mortality. Second, primary care physicians’ widespread misconceptions about overweight BMI should be rectified. Third, the evidence basis for other potential risks or benefits of overweight BMI should be rigorously examined and incorporated appropriately into health communication.

## Background

About 30% of adults in the United States are overweight (body mass index [BMI] 25–29.9 kg/m^2^), and another 40% have obesity (BMI ≥ 30 kg/m^2^). The prevalence of overweight and obesity continues to rise worldwide, especially in low- and middle-income countries. Reducing overweight and obesity has been a longstanding focus of public health messaging and physician–patient interactions. Conveying evidence-based recommendations to the public about potentially modifiable risk factors, including BMI, is a commendable objective. However, as we will describe, current communication often substantially overstates all-cause mortality risks associated with the range of BMIs classified as “overweight” but not “obese” (i.e., 25–29.9 kg/m^2^). Health communication that inaccurately conveys current evidence may do more harm than good. Although we focus on all-cause mortality as one important health outcome, other outcomes such as cardiovascular risk are also relevant to public health communication. Discrepancies between evidence and communication regarding mortality raise the question of whether similar discrepancies exist for other health outcomes.

### Current health communication

Current public health recommendations describe both overweight and obesity as risk factors for mortality and other health conditions. The World Health Organization states that “worldwide, at least 2.8 million people die each year as a result of being overweight or obese” [1]. The Centers for Disease Control states that “people who have overweight and obesity…are at increased risk for many serious diseases and health conditions,” specifically listing “all-causes of death” first among these [2]. Risks are conveyed similarly in communication from national and international health authorities to clinicians: clinical guidelines by the American College of Cardiology and American Heart Association Task Force recommend that physicians “advise overweight and obese adults that the greater the BMI, the greater the risk of CVD, type 2 diabetes, and all-cause mortality” [3]. Accordingly, a recent survey of primary care physicians (PCPs) in the United States found that a large majority (90%) believed that overweight BMI (exclusive of obesity) increases mortality risk, and they typically believed that the increased risk is substantial (a 25% to 59% increase) [4]. In a small study of 14 PCPs in Scotland, every participating PCP agreed that “normal weight is important for health” [5]. PCPs believe that they play an essential role in managing overweight and obesity, and it is typically PCPs rather than patients themselves who initiate conversations about BMI [6]. Thus, their risk perceptions may substantially affect their communication with patients.

### Current empirical evidence

Currently available evidence indeed suggests that obesity (as well as underweight) is associated with increased all-cause mortality, although much of the literature has methodological limitations, including possible confounding and, when BMI is self-reported, measurement error [7, 8]. On the other hand, the current evidence overall suggests that overweight BMI (exclusive of obesity) is not associated, or is only modestly associated, with mortality when compared to normal BMI. Among studies that do indicate an association, the direction is inconsistent: in many studies, overweight BMI is associated with modestly reduced, rather than increased, mortality. Two prominent meta-analyses in general populations reported modest associations in opposite directions for the overweight category (hazard ratio *HR* = 0.94 versus *HR* = 1.11 [9, 10]). Another meta-analysis suggested that in older adults, overweight BMI may be associated with modestly reduced mortality [11]. Overall, clinical guidelines summarize the empirical evidence thus: “The current category for overweight (BMI 25.0 to 29.9 kg/m^2^) is not associated with elevated risk of all-cause mortality, but a BMI at or above the current cutpoint for obesity (BMI ≥ 30 kg/m^2^) is associated with an elevated risk of all-cause mortality, compared with normal weight.” [3] These are the same clinical guidelines that nevertheless recommend advising patients that higher BMIs increase the risk of mortality, with no suggested distinction between overweight and obese BMIs.

While these meta-analyses and many of their constituent studies had important methodologic limitations [7, 8], individual studies with stronger designs have yielded similar findings. We discuss some examples of large, longitudinal studies with fairly robust control of confounding by variables such as existing health conditions, smoking, and prior BMI [7, 8]. The studies we discuss are merely examples and not an exhaustive list; indeed, a new evidence synthesis with more stringent methodological inclusion criteria would be informative [12].

To help address the limitations of self-reporting and of BMI as a standalone measure of adiposity, a study of 369,752 participants in the UK Biobank assessed both lab-measured BMI and direct measures of adiposity, such as fat mass [13]. On average across age groups (ranging from 45 to 85 years), overweight was not associated with mortality, though there may have been protective effects for younger participants [13]. A study of 15,792 participants in the Atherosclerosis Risk in Communities Study used marginal structural models to better control for time-varying confounding, including confounding induced by prior BMI [14]. Overweight was protective among ever-smokers (incidence rate ratio [*IRR*] = 0.77; 95% confidence interval [0.68, 0.88]) and was not meaningfully associated with mortality among never-smokers (*IRR* = 1.06 [0.80, 1.41]). Another study of 14,345 men in the Aerobics Center Longitudinal Study was unusual in its ability to control for changes in cardiorespiratory fitness over time, along with numerous other confounders. This study found that, with appropriate control for these variables, BMI gain (vs. BMI loss) was not associated with mortality (*HR* = 1.03 [0.87, 1.23]). Other studies attempt to address confounding by existing health conditions and prior BMI by considering participants’ maximum BMI, rather than their BMI at a fixed point in time. One such study, involving 225,072 participants in the Nurses’ Health Studies I and II and the Health Professionals Follow-Up Study, assessed associations of participants’ maximum BMIs over 16 years with mortality [15]. This study reported that overweight was associated with increased mortality, although the effect size was again modest (*HR* = 1.06 [1.03, 1.08]). Corroborating the epidemiologic evidence from these studies and the meta-analyses, randomized trials have suggested that for patients with overweight or obese BMI, weight loss does not clearly improve either morbidity or mortality, although there may be a benefit for patients with morbid obesity [16, 17].

## Conclusions

The question of whether BMIs currently classified as “overweight” are a risk factor for all-cause mortality is not fully resolved given methodological limitations. However, as noted above, the strongest available evidence typically does not suggest clinically meaningful associations of overweight with increased mortality and often instead suggests modest associations in the opposite direction. We would therefore make the following recommendations regarding communication about overweight BMI and mortality:

- *Recommendation 1*. Top-line recommendations to the public and to health practitioners who inform the public should carefully distinguish overweight BMI from obese BMIs. At this time, BMIs categorized as “overweight” should no longer be described as contributing to all-cause mortality.
- *Recommendation 2*. Continuing education and clinical practice guidelines should address primary care physicians’ widespread misconception that overweight BMIs substantially increase mortality risk, because these conceptions far exceed empirical estimates and may distort physicians’ recommendations to patients.

A holistic, evidence-based approach to public health communication would also consider other outcomes in addition to mortality. Despite the null or weak associations of overweight BMI with all-cause mortality, it is possible that overweight BMI could affect cause-specific mortality, such as cardiovascular mortality, or could affect disease burden and quality of life. Additionally, for some individuals, becoming overweight could ultimately lead to becoming obese, which is a greater health concern. Indeed, over 90% of individuals gain weight in midlife, and among individuals who are overweight in young adulthood, an estimated 46% of men and 40% women become obese over the next 18 years [18]. An evidence-based approach would allow health practitioners to better tailor communication based on individual patient characteristics (for example, for some patients, the dialogue may stress the importance of not gaining more weight rather than on losing weight). For these reasons, we make a third recommendation:

- *Recommendation 3*. The evidence basis for other potential risks or benefits of overweight BMI (e.g., cardiovascular complications or development of obesity) should be rigorously examined and incorporated appropriately into health communications from health authorities to health practitioners so that the latter can, in turn, counsel individual patients appropriately.

For the 30% of adults in the United States who are currently classified as “overweight” but not “obese,” addressing these three points could potentially reduce undue medicalization, stress, and resources spent on weight-loss efforts that may not always be medically indicated.

Over time, a larger body of methodologically rigorous evidence on BMI and health outcomes, including mortality, will emerge. At that point, it may be time to revisit whether the BMI categories themselves could be redefined to better align with concrete health risks, including but not limited to mortality. The current designation of BMIs greater than 25 as overweight or obese originated with a 1995 report by the World Health Organization, which acknowledged that “there is no agreement about cut-off points for the percentage of body fat that constitutes obesity” [16]. If BMI is to be used as an evidence-based marker of health risks, the categories might ultimately need to be redefined based on the totality of rigorous evidence. Given that optimal BMI may differ across populations, categories and recommendations may need to differ by characteristics such as race, age, and sex.

## Data Availability

Not applicable (no data were collected or analyzed).

## Abbreviations

BMI: Body mass index
HR: Hazard ratio
